# Health-related quality of life in patients with metastatic colorectal cancer treated with panitumumab in first- or second-line treatment

**DOI:** 10.1038/bjc.2011.409

**Published:** 2011-10-11

**Authors:** L Bennett, Z Zhao, B Barber, X Zhou, M Peeters, J Zhang, F Xu, J Wiezorek, J-Y Douillard

**Affiliations:** 1 RTI Health Solutions, 3040 East Cornwallis Road, Post Office Box 12194, Research Triangle Park, NC 22709-2194, USA; 2 Amgen Inc., One Amgen Center Drive, Thousand Oaks, CA 91320-1799, USA; 3 Department of Oncology, University Hospital Antwerp, Wilrijkstraat 10, Edegem, Antwerp B-2650, Belgium; 4 Department of Medical Oncology, Centre René Gauducheau, Boulevard J Monod, St Herblain 44805, France

**Keywords:** colorectal cancer, panitumumab, quality of life

## Abstract

**Background:**

Panitumumab in combination with chemotherapy was evaluated in two pivotal clinical trials in first- and second-line treatment of metastatic colorectal cancer (mCRC), respectively. This analysis compared the health-related quality of life (HRQoL) of patients with or without panitumumab in the two trials.

**Methods:**

Patients with mCRC were randomised to FOLFOX (first-line trial) or FOLFIRI (second-line trial)±panitumumab. The EuroQoL 5-Dimensions Health State Index (EQ-5D HSI) and Visual Analogue Scale (EQ-5D VAS) were assessed at baseline and monthly follow-up until disease progression. Patients with wild-type *KRAS* mCRC with baseline and post-baseline HRQoL scores were included. Difference in change from baseline between treatment groups was evaluated using linear mixed and pattern-mixture models.

**Results:**

In the first-line trial, 576 patients with wild-type *KRAS* mCRC (284 panitumumab+FOLFOX4 and 292 FOLFOX4 alone) were included in the HRQoL analyses. In the second-line trial, 530 patients with wild-type *KRAS* mCRC were included in these analyses (263 panitumumab+FOLFIRI and 267 FOLFIRI alone). There was no significant difference in the change in EQ-5D HSI and VAS scores between treatment groups in either trial.

**Conclusion:**

The addition of panitumumab to FOLFOX4 or FOLFIRI in first- or second-line treatment of wild-type *KRAS* mCRC significantly improved progression-free survival without compromising HRQoL.

Recent advances in the development of treatments for metastatic colorectal cancer (mCRC) have led to improved outcomes in this group of patients (Hurwitz *et al*, 2004; Amado *et al*, 2008; Kabbinavar *et al*, 2008; Karapetis *et al*, 2008; Saltz *et al*, 2008; Bokemeyer *et al*, 2009; Van Cutsem *et al*, 2009; Tebbutt *et al*, 2010). Panitumumab, a fully human monoclonal antibody (mAb) targeting the epidermal growth factor receptor (EGFR), originally demonstrated improved progression-free survival (PFS) in combination with best supportive care (BSC) *vs* BSC alone in patients with wild-type *KRAS* chemorefractory mCRC (Amado *et al*, 2008). Further evaluation of the role of panitumumab in patients with wild-type *KRAS* mCRC has recently shown that its PFS benefits extend to earlier lines of treatment when used in combination with chemotherapy (Douillard *et al*, 2010; Peeters *et al*, 2010). In a multicentre, randomised, phase III trial, the combination of panitumumab and FOLFOX4 significantly increased PFS compared with FOLFOX4 alone in the first-line treatment of patients with wild-type *KRAS* mCRC (median PFS 9.6 *vs* 8.0 months; hazard ratio (HR)=0.80; 95% confidence interval (CI): 0.66–0.97; *P*=0.02) (Douillard *et al*, 2010). In another multicentre, randomised, phase III trial, involving patients that had received one prior systemic therapy, the combination of panitumumab and FOLFIRI also significantly increased median PFS (5.9 *vs* 3.9 months; HR=0.73; 95% CI: 0.59–0.90; *P*=0.004) (Peeters *et al*, 2010) compared with FOLFIRI alone in patients with wild-type *KRAS* mCRC. Furthermore, panitumumab in combination with chemotherapy has consistently demonstrated a non-significant trend toward improved overall survival (OS) in both first- and second-line treatment of wild-type *KRAS* mCRC. Among first-line patients, an increase of 4.2 months in median OS was observed among patients receiving panitumumab+FOLFOX4 over those receiving FOLFOX4 alone (*P*=0.072) (Douillard *et al*, 2010). A 2-month increase in median OS was observed in second-line patients treated with panitumumab+FOLFIRI compared with those treated with FOLFIRI alone (*P*=0.12) (Peeters *et al*, 2010). Data from both phase III trials were analysed by *KRAS* status prospectively with >90% ascertainment.

In metastatic disease, in addition to delaying disease progression, maintenance of health-related quality of life (HRQoL) – a measure of how a disease or its treatment affects a persons' physical, emotional and social well-being (Cella, 1995) – is a particularly important aim of treatment (Van Cutsem *et al*, 2010). Panitumumab is associated with a well-defined adverse event profile (in particular, dermatologic toxicities, a characteristic side effect of EGFR inhibitors (Agero *et al*, 2006)), and it is important to understand how the impact of such toxicities weigh against the benefits of the drug. The EuroQoL 5-Dimensions (EQ-5D) is a validated, standardised and widely used instrument that can be used to measure HRQoL (Dolan, 1997). The evaluation of changes in HRQoL using the EQ-5D was a tertiary objective in both the first- and second-line phase III clinical trials of panitumumab. The aims of the current analysis were to evaluate the impact of the addition of panitumumab to FOLFOX4 or FOLFIRI treatment on HRQoL in the aforementioned first- and second-line studies (Douillard *et al*, 2010; Peeters *et al*, 2010). As panitumumab treatment has an increased risk of skin toxicity, a common adverse event with anti-EGFR agents, we also sought to assess the correlation between HRQoL and severity of skin toxicity.

## Patients and methods

### Patients and study design

The design and patient population for these two phase III, open-label, randomised, controlled trials (NCT00364013; NCT00339183) have been described in detail in previous publications (Douillard *et al*, 2010; Peeters *et al*, 2010). The protocols were approved by the ethics committees at participating sites. All patients signed informed consent before any study-related procedures were performed.

Briefly, the first-line trial compared the efficacy and safety of the combination of panitumumab+FOLFOX4 with FOLFOX4 alone in patients with previously untreated mCRC (Douillard *et al*, 2010), while the second-line trial compared the efficacy and safety of the combination of panitumumab+FOLFIRI with FOLFIRI alone in patients with previously treated mCRC (Peeters *et al*, 2010). Patients were randomly assigned 1 : 1 to panitumumab+chemotherapy *vs* chemotherapy alone in both trials. In the first-line trial, randomisation was stratified by geographic region, and Eastern Cooperative Oncology Group (ECOG) performance status. In the second-line trial, randomisation was stratified by prior oxaliplatin exposure for mCRC, prior bevacizumab exposure for mCRC and ECOG performance status. Panitumumab 6.0 mg kg^–1^ was administered without pre-medication by intravenous infusion every 2 weeks on day 1 before the appropriate chemotherapy (FOLFOX4 or FOLFIRI), while patients randomised to the chemotherapy group received FOLFOX4 or FOLFIRI alone. Treatment was administered until disease progression or unacceptable toxicity.

### HRQoL measurements

Health-related quality of life was measured using the EQ-5D Health State Index (EQ-5D HSI) and the EQ-5D Visual Analogue Scale (EQ-5D VAS). Assessments were taken at baseline and monthly until disease progression, and then once at the 4-week safety follow-up visit. Patients who withdrew from the study treatment before disease progression (e.g., because of unacceptable toxicities) were encouraged to complete assessments every 8 weeks (±1 week) until disease progression and at the safety follow-up visit.

The EQ-5D HSI assesses health across five dimensions that include mobility, self-care, usual activities, pain or discomfort, and anxiety or depression. Each dimension has three possible outcomes (no problems, moderate problems and extreme problems), with a total score for EQ-5D HSI ranging from −0.594 to 1 (Dolan, 1997) using published tariffs developed for the United Kingdom. The minimal clinically important difference (MCID) for the EQ-5D HSI has been estimated as a change in score of ⩾0.08 (Pickard *et al*, 2007a). The EQ-5D VAS provides an assessment of current health status on a vertical scale of 0–100, with 0 representing ‘worst imaginable health’ and 100 representing ‘best imaginable health’. The MCID for the EQ-5D VAS has been estimated as a change in score of ⩾7 (Pickard *et al*, 2007a). The analysis of the EQ-5D HSI and VAS sought to estimate in both trials, in patients with wild-type *KRAS* mCRC, the average difference in effect of panitumumab+FOLFOX4 (or +FOLFIRI) compared with FOLFOX4 alone (or FOLFIRI alone); and the correlation between skin toxicity and change from baseline in EQ-5D HSI and EQ-5D VAS scores in patients treated with panitumumab. Skin toxicities were reported as adverse events that were collected during the treatment and safety follow-up phases and were graded using the National Cancer Institute Common Terminology Criteria for Adverse Events version 3.0 with modifications for specific skin and nail toxicities (National Cancer Institute, 2006).

### Statistical analysis

The analysis was performed separately for the two clinical trials. In each trial, the intent-to-treat (ITT) population included all randomised subjects with wild-type *KRAS* mCRC, regardless of treatment received. In the current analysis, we included subjects in the ITT population who had a baseline and at least one post-baseline HRQoL assessment for each score analysed before disease progression by central assessment.

#### Linear mixed effects model

The same statistical analyses were conducted separately for each of the two studies. Changes in EQ-5D HSI and EQ-5D VAS scores from baseline for treatment effects were analysed over time using linear mixed models for repeated measures with intercept and slope for study week as random effects. The fixed effects in the initial models included explanatory variables for study treatment arm, study week, and the interaction between treatment arm and study week. Each mixed model also included covariates for baseline EQ-5D score, ECOG performance statues (0 or 1 *vs* 2), geographic region (western Europe, Canada, the USA and Australia *vs* rest of world), and three corresponding treatment-by-covariates interaction terms. For analysis of the second-line trial, two additional baseline covariates – prior bevacizumab exposure for mCRC (yes *vs* no) and prior oxaliplatin for mCRC (yes *vs* no), and two corresponding treatment-by-covariates interaction terms were included in the initial models. Backward selection was used to eliminate interaction terms with baseline variables if not significant at the 0.05 level, but all main effects and the time-by-treatment interaction remained in the final model regardless of significance. Treatment-specific least squares mean (LSM) estimates of average change in each outcome from baseline, along with 95% CI, were calculated.

#### Pattern mixture model

To evaluate the effect of study attrition on the estimate of treatment differences, a sensitivity analysis was performed using pattern mixture models to incorporate information on patterns of missing data. Assuming intermittent missing data occurred at random, patients were classified into two pattern groups: early dropout (defined as dropout on or before week 28 (week 20) in the first-line (second-line) study) and late dropout/completer (those who either dropped out after week 28 (week 20) or completed all assessments in the first-line (second-line) study). A different cut-off week, at around the 75% dropout rate in each trial, was used to define early *vs* late dropout for the two trials to reflect the difference in study duration between the two trials. Within each dropout group, LSM estimates of average change in each outcome from baseline, along with 95% CI, were calculated.

#### Impact of skin toxicity on HRQoL

The impact of skin toxicity on the changes in EQ-5D HSI and EQ-5D VAS scores from baseline in patients receiving panitumumab was estimated using a linear mixed effects model. Patients were divided into three groups based on the level of skin toxicity reported: no/mild toxicity (grade 0 or 1), moderate toxicity (grade 2) and severe toxicity (grade 3+). This analysis was outcome-by-outcome and evaluated an association not cause–effect. LSM estimates of average change from baseline within each subgroup, along with 95% CI, were calculated.

## Results

### Demographics

In the first-line study, there were 656 patients with wild-type *KRAS* mCRC (Douillard *et al*, 2010). Of these, 576 patients (284 received panitumumab+FOLFOX4 and 292 received FOLFOX4 alone) were included in the HRQoL analysis, representing 87.8% of the overall wild-type *KRAS* population. Specifically, 279 and 289 patients receiving panitumumab+FOLFOX4 and FOLFOX4 alone, respectively, were eligible for inclusion in the EQ-5D HSI analysis; while the EQ-5D VAS analysis included 278 patients receiving panitumumab plus FOLFOX4 and 285 receiving FOLFOX4 alone.

In the second-line study, 597 patients with wild-type *KRAS* tumours were included (Peeters *et al*, 2010). Of these, 530 patients (263 received panitumumab+FOLFIRI and 267 received FOLFIRI) were included in the HRQoL analysis, representing 88.8% of the overall wild-type *KRAS* population. For the EQ-5D HSI analysis, 262 and 265 patients from the panitumumab+FOLFIRI arm and the FOLFIRI alone arm, respectively, were included; and the EQ-5D VAS score analysis included 257 patients from the panitumumab+FOLFIRI arm and 259 patients from the FOLFIRI alone arm. Baseline demographics and clinical characteristics, including EQ-5D HSI and EQ-5D VAS scores at baseline, were generally well-balanced across treatment groups in both studies (Table 1) and were similar to those reported in the overall patient populations (Douillard *et al*, 2010; Peeters *et al*, 2010).

### First-line trial

#### Changes in scores from baseline – linear mixed model

The mean baseline score for the EQ-5D HSI was 0.778 for the panitumumab+FOLFOX4 arm and 0.756 for the FOLFOX4 alone arm. Mean baseline EQ-5D VAS scores were 74.1 and 70.1, respectively (Table 1). According to the mixed model for change from baseline score, there was a slight within-group improvement (which was statistically significant, but not clinically meaningful) in the EQ-5D HSI score in the panitumumab+FOLFOX4 and FOLFOX4 alone groups (Table 2a). The estimate (LSM of the change from baseline was 0.022 (95% CI: 0.003–0.041) in the panitumumab+FOLFOX4 group and 0.027 (95% CI: 0.008–0.046) in the FOLFOX4 alone group. The difference between the two treatment arms was −0.005 (95% CI: −0.032–0.022), which was not statistically significant or clinically meaningful. Similar results were observed in EQ-5D VAS scores (Table 2a). The estimated LSM of the change from baseline for EQ-5D VAS was 1.228 (95% CI: −0.378–2.834) in the panitumumab+FOLFOX4 group and 1.881 (95% CI: 0.275–3.487) in the FOLFOX4 alone group. The difference between the two treatment arms was −0.653 (95% CI: −2.925 to 1.618), which was not statistically significant or clinically meaningful.

#### Pattern mixture model (sensitivity analysis)

For each outcome, missing data were categorised into early and late dropout/completer patterns. A summary of the dropout pattern is provided in Table 3. A significantly greater proportion of patients in the panitumumab+FOLFOX4 group were categorised as late dropouts/completers compared with the FOLFOX4 alone group. The difference was around 10% in both the EQ-5D HSI and VAS analysis sets (*P*=0.010 and *P*=0.050, respectively) (Table 3). These data indicated that the dropout patterns were different between the two treatment groups. The missing data were considered to be non-random and treatment effects were evaluated separately for those patients who did and those who did not drop out early. Within each dropout group, LSM estimates of average change in EQ-5D HSI and VAS from baseline, along with 95% CI, are provided in Table 4a. There was a statistically significant but not clinically meaningful improvement in the EQ-5D HSI (panitumumab+FOLFOX4: 0.058; 95% CI: 0.032–0.084; FOLFOX4 alone: 0.062; 95% CI: 0.033–0.091) and EQ-5D VAS (panitumumab+FOLFOX4: 4.008; 95% CI: 1.677–6.339; FOLFOX4 alone: 4.392; 95% CI: 1.887–6.898) scores within each treatment arm in the late dropout/completer group. This pattern was absent in the early dropout group. No statistically significant or clinically meaningful difference was observed in either the early dropout or the late dropout/completer group between the two treatment arms in both the EQ-5D HSI and EQ-5D VAS score (Table 4a). Therefore, the pattern mixture analyses estimating average differences in change from baseline score for the EQ-5D HSI and EQ-5D VAS for both early and late dropout/completers resulted in a similar outcome (no treatment difference) to the linear mixed models for all patients with wild-type *KRAS* mCRC.

#### Correlation between skin toxicity and HRQoL

On the basis of the mixed model of change from baseline score, LSM estimates of average change from baseline along with 95% CI were calculated for patients by severity level of skin toxicity. Baseline mean scores and LSM estimates of changes from baseline for the EQ-5D index-based scores and VAS scores associated with each category of skin toxicity are reported in Table 5a. The difference in change from baseline EQ-5D HSI and EQ-5D VAS score between any two of the three groups (no/mild, moderate or severe skin toxicity) was not statistically significant or clinically meaningful (Table 5b).

### Second-line trial

#### Changes in scores from baseline – linear mixed model

The mean baseline score for the EQ-5D HSI was 0.769 in the panitumumab+FOLFIRI group and 0.762 in the FOLFIRI alone arm. Mean baseline EQ-5D VAS scores were 73.3 and 71.9, respectively. According to the mixed model of change from baseline score, the estimated LSM of the change from baseline EQ-5D HSI was −0.024 (95% CI: −0.045 to −0.003) in the panitumumab+FOLFIRI group and +0.000 (95% CI: −0.021 to 0.022) in the FOLFIRI alone group (Table 2b). The changes from baseline in both arms were not clinically meaningful. The difference between the two treatment arms was −0.024 (95% CI: −0.054 to 0.006), and was not statistically significant or clinically meaningful. Similar results were observed in EQ-5D VAS; the estimated LSM of the change from baseline was −1.438 (95% CI: −2.838 to −0.037) in the panitumumab+FOLFIRI group and −0.172 (95% CI: −1.620 to 1.275) in the FOLFIRI alone group (Table 2b). The difference between the two treatment arms was −1.266 (95% CI: −3.280 to 0.749), which was not statistically significant or clinically meaningful.

#### Pattern mixture model (sensitivity analysis)

Similar to the first-line trial, compared with patients in the FOLFIRI alone arm, patients in the panitumumab+FOLFIRI arm were more likely be categorised as late dropout/completers. The difference in the late dropout rate between the two treatment arms was around 10% in both EQ-5D HSI and VAS analysis sets (*P*=0.017 and 0.008, respectively) (Table 3). Thus, there were different dropout patterns between the two treatment arms and treatment effects were evaluated separately for those patients who did and those who did not drop out early. For each outcome, missing data were categorised into early and late dropout/completer patterns. Within each dropout group, LSM estimates of average change in EQ-5D HSI and EQ-5D VAS from baseline, along with 95% CI, are provided in Table 4b. There was no statistically or clinically meaningful change in the EQ-5D HSI in both treatment groups (panitumumab+FOLFIRI: 0.023; 95% CI: −0.008 to 0.053; FOLFIRI alone: 0.025; 95% CI: −0.011 to 0.060) in late dropout/completers. The change from baseline for the EQ-5D VAS was 1.575 (95% CI: −0.477 to 3.627) for the panitumumab+FOLFIRI group and 3.310 (95% CI: 0.875–5.745) for FOLFIRI alone group in the late dropout/completer group. In the early dropout group, the change from baseline scores was generally worse than those in the late dropout/completer group. The pattern mixture analyses estimating average differences between treatments in change from baseline score for the EQ-5D HSI and EQ-5D VAS scores for both early and late dropout/completers resulted in generally similar results as the linear mixed models for all patients with wild-type *KRAS* mCRC.

#### Correlation between skin toxicity and HRQoL

Similar to the analyses of the first-line study, the change from baseline EQ-5D HSI and EQ-5D VAS score was estimated for patients by skin toxicity severity level. Baseline mean scores and LSM estimates of changes from baseline for the EQ-5D index-based scores and VAS scores associated with each category of skin toxicity were reported in Table 5a. The difference in change from baseline for both EQ-5D HSI and EQ-5D VAS between any two of the three groups (no/mild, moderate or severe skin toxicity) was not statistically significant or clinically meaningful (Table 5b).

## Discussion

As the possible options for the treatment of mCRC have expanded and survival has increased, patient HRQoL has become an increasingly important treatment outcome, especially as treatment aims in this setting are generally palliative rather than curative (Byrne *et al*, 2007). The addition of panitumumab to FOLFOX4 as a first-line treatment, or to FOLFIRI as a second-line treatment significantly increases PFS in patients with wild-type *KRAS* mCRC (Douillard *et al*, 2010; Peeters *et al*, 2010). In our analyses of the EQ-5D assessments made during these phase III studies, there were no statistically significant or clinically meaningful overall differences in the change in HRQoL in patients treated with panitumumab+chemotherapy compared with those treated with chemotherapy alone. This suggests that the addition of panitumumab to chemotherapy regimens as a first- or second-line treatment of patients with wild-type KRAS mCRC provides improvements in PFS without compromising HRQoL. Additionally, there were significantly more patients who dropped out late or completed the study in the group receiving panitumumab than the group receiving chemotherapy only in both first- and second-line trials.

When used in addition to BSC in patients with chemorefractory wild-type KRAS mCRC, panitumumab has been reported to provide better control of symptoms and maintenance of HRQoL compared with BSC alone (Odom *et al*, 2011). Thus, our results extend our understanding of the impact of panitumumab on HRQoL to earlier lines of mCRC treatment. In contrast to the findings in the chemorefractory setting, we did not find a difference in the change in HRQoL between treatment arms in our analyses. Generally, HRQoL is relatively high at baseline in first-line mCRC patients, reflecting the fact that patients are often predominantly asymptomatic when diagnosed with mCRC (0.778 for first-line patients from this study *vs* 0.845 for general population (Petrou and Hockley, 2005)). This indicates that the realistic treatment goal in first-line mCRC patients is to maintain rather than to improve HRQoL. In second-line mCRC patients, the baseline HRQoL in our study was still relatively high; this may be because these patients have been enrolled in an experimental clinical trial and may be more hopeful of achieving a good outcome: this, in turn, may impact positively on their baseline HRQoL. Thus, the realistic treatment goal in this group of patients is also to maintain HRQoL. The HRQoL for patients failing standard chemotherapies and seeking third-line treatment tends to deteriorate rapidly, however, and hence this is where HRQoL benefits with panitumumab have been demonstrated (Odom *et al*, 2011).

Our findings regarding the impact of panitumumab on HRQoL in the first- and second-line treatment of mCRC are consistent with those observed in studies of cetuximab and bevacizumab (a mAb targeting the vascular endothelial growth factor) in this setting. There was neither a clinically meaningful nor statistically significant difference in HRQoL in patients treated with cetuximab+FOLFIRI *vs* FOLFIRI alone as a first-line treatment for wild-type *KRAS* mCRC (Folprecht *et al*, 2009). Similar data have also been reported in three studies of bevacizumab. In first-line mCRC patients, except one measure in one phase II study, the time to deterioration in HRQoL did not differ significantly between patients receiving bevacizumab combined with 5-fluorouracil(5-FU)/leucovorin, irinotecan/5-FU/leucovorin or capecitabine compared with the use of these chemotherapies alone (Kabbinavar *et al*, 2008; Tebbutt *et al*, 2010).

Owing to the perceived impact of skin toxicity (which occurs in up to 90% of patients treated with panitumumab) on patient-reported outcomes, we further analysed the impact of skin toxicities on HRQoL. Our results indicated that the severity level of skin toxicity was independent of HRQoL. Therefore, regardless of the severity of skin toxicity, patients treated with panitumumab maintained a similar HRQoL. Severe skin toxicity associated with panitumumab has been reported to correlate with improved survival (Siena *et al*, 2007; Douillard *et al*, 2010; Peeters *et al*, 2010) and this is of note as HRQoL is likely affected by both efficacy benefits and skin toxicity, such that the net result in our study was that panitumumab did not have a negative impact on HRQoL.

In oncology trials, it is common for substantial amounts of patient-reported data to be missing. As the disease progresses and disease symptoms increase, the burden of completing patient-reported outcome questionnaires increases. These missing data cause methodological challenges in analysing HRQoL data. We employed linear mixed models, which is a superior approach compared with the traditional analysis of covariance models used with imputation of missing data, such as the last observation carried forward method (Siddiqui *et al*, 2009). Furthermore, in both studies, there was a significantly increased rate of early dropout in the chemotherapy only arm compared with the panitumumab+chemotherapy arm. As a sensitivity analysis, we used the pattern mixture models to control for the impact of non-random missing data as well as different patterns of missing data between the treatment arms. The overall results were similar using both models.

A few limitations of the analysis presented here should be noted. First, EQ-5D was chosen over other HRQoL measures because it has been validated and commonly used in mCRC studies (Pickard *et al*, 2007b); utility can be generated from EQ-5D index scores for cost-effectiveness analysis; and it is easy to administer yet has a comparable responsiveness to that of a disease-specific HRQoL (EORTC QLQ C-30 scales) (Krabbe *et al*, 2004). However, for the purpose of assessing the impact of skin toxicities on HRQoL, the existing brief skin-specific HRQoL measures are better instruments and might have been administered in conjunction with the EQ-5D. Second, a large majority of the patients in these trials had ECOG scores of 0 or 1, so the conclusions reported here may not fully represent the impact of panitumumab treatment on HRQoL in a broader population of patients with mCRC. The use of other EGFR inhibitors has been shown to impact HRQoL in other studies (Andreis *et al*, 2010). Additionally, the EQ-5D is not a direct measure of the impact of skin toxicity on HRQoL, and this may explain the lack of statistically significant differences in EQ-5D across levels of skin toxicity. It should also be noted that differences in EQ-5D scores smaller than those cited (e.g., Pickard *et al*, 2007a) may be important, so the interpretation of related findings needs to be cautious. Next, for fitting the pattern mixture models, we assumed that any intermittent missing data before dropout or study completion was missing at random. If the missing value happened because of treatment-related toxicity or side effects, which were not included as covariates in the models, this assumption may not be appropriate.

In conclusion, our analysis further extends our understanding of the recently reported benefits of panitumumab in combination with FOLFOX4 or FOLFIRI as a first- or second-line treatment for wild-type *KRAS* mCRC (Douillard *et al*, 2010; Peeters *et al*, 2010). In spite of anti-EGFR-related skin toxicity, the overall HRQoL for patients receiving panitumumab+chemotherapy was not adversely affected. Therefore, the improvements in PFS observed with panitumumab are obtained without compromising patients' HRQoL.

## Figures and Tables

**Table 1:** Baseline demographics and clinical characteristics of patients with wild-type *KRAS* metastatic colorectal cancer included in the EQ-5D HSI and EQ-5D VAS from two trials of panitumumab

	**First-line trial**		**Second-line trial**	
	**Panitumumab + FOLFOX4 (*n*=284)**	**FOLFOX4 (*n*=292)**	**Panitumumab + FOLFIRI (*n*=263)**	**FOLFIRI (*n*=267)**
Male, n (%)	189 (66.5%)	186 (63.7%)	159 (60.5%)	171 (64.0%)
Age, mean years (s.d.)	60.5 (10.5)	60.1 (11.3)	60.1 (10.1)	60.6 (10.1)
White race, n (%)	257 (90.5%)	271 (92.8%)	255 (97.0%)	253 (94.8%)
				
*Region,* n (%)				
Australia, USA, western Europe, Canada	166 (58.5%)	161 (55.1%)	162 (61.6%)	163 (61.0%)
Rest of world	118 (41.5%)	131 (44.9%)	101 (38.4%)	104 (39.0%)
				
*ECOG performance status,* n (%)				
0–1	270 (95.1%)	276 (94.5%)	253 (96.2%)	255 (95.5%)
⩾2	14 (4.9%)	16 (5.5%)	10 (3.8%)	12 (4.5%)
				
*Primary tumour type,* n (%)				
Colon	183 (64.4%)	192 (65.8%)	160 (60.8%)	171 (64.0%)
Rectal	101 (35.6%)	100 (34.2%)	103 (39.2%)	96 (36.0%)
				
*Site of metastatic disease,* n (%)				
Liver only	53 (18.7%)	48 (16.4%)	56 (21.0%)	46 (17.5%)
Liver+other	193 (68.0%)	199 (68.2%)	169 (63.3%)	178 (67.7%)
Other only	37 (13.0%)	44 (15.1%)	40 (15.0%)	39 (14.8%)
Missing or unknown	1 (0.4%)	1 (0.3%)	2 (0.7%)	0 (0.0%)
				
*Prior therapy,* n (%)				
Oxaliplatin	NA	NA	175 (66.5%)	172 (64.4%)
Bevacizumab	NA	NA	49 (18.6%)	55 (20.6%)
				
*Baseline EQ-5D HSI*				
Mean (s.d.)	0.778 (0.247)	0.756 (0.244)	0.769 (0.230)	0.762 (0.252)
				
*Baseline EQ-5D VAS*				
Mean (s.d.)	74.1 (19.3)	70.1 (20.8)	73.3 (17.3)	71.9 (18.8)

Abbreviations: ECOG=Eastern Cooperative Oncology Group; EQ-5D HSI=EuroQoL 5-Dimensions Health State Index; EQ-5D VAS= EQ-5D Visual Analogue Scale; FOLFIRI=fluorouracil, leucovorin and irinotecan; FOLFOX=fluorouracil, leucovorin and oxaliplatin; NA=not applicable; s.d.=standard deviation.

**Table 2:** LSM differences in change from baseline in the EQ-5D HSI and EQ-5D VAS scores between (**A**) panitumumab + FOLFOX4 *vs* FOLFOX4-only and (**B**) panitumumab + FOLFIRI *vs* FOLFIRI-only, using linear mixed models

**(A)**						
	**EQ-5D HSI**			**EQ-5D VAS**		
	**Panitumumb + FOLFOX4 (*n*=279)**	**FOLFOX4 (*n*=289)**	**Difference**	**Panitumumb + FOLFOX4 (*n*=278)**	**FOLFOX4 (*n*=285)**	**Difference**
LSM	0.022	0.027	−0.005	1.228	1.881	−0.653
(95% CI)	(0.003, 0.041)	(0.008, 0.046)	(−0.032, 0.022)	(−0.378, 2.834)	(0.275, 3.487)	(-2.925, 1.618)
						
**(B)**						
	**EQ-5D HIS**			**EQ-5D VAS**		
	**Panitumumb + FOLFIRI (*n*=262)**	**FOLFIRI (*n*=265)**	**Difference**	**Panitumumb + FOLFIRI (*n*=257)**	**FOLFIRI (*n*=259)**	**Difference**
LSM difference	−0.024	0.000	−0.024	−1.438	−0.172	−1.266
(95% CI)	(−0.045, −0.003)	(−0.021, 0.022)	(−0.054, 0.006)	(−2.838, −0.037)	(−1.620, 1.275)	(−3.280, 0.749)

Abbreviations: CI=confidence interval; EQ-5D=EuroQoL-5 dimensions; FOLFIRI=fluorouracil, leucovorin and irinotecan; FOLFOX=fluorouracil, leucovorin and oxaliplatin; HSI=Health State Index; LSM=least squares mean; VAS=Visual Analogue Scale.

**Table 3:** Summary of dropout pattern by treatment in first- and second-line trials

	**First-line trial**			**Second-line trial**		
**Instrument/ pattern**	**Panitumumab + FOLFOX4 *n* (%)**	**FOLFOX4 alone *n* (%)**	***P***	**Panitumumab + FOLFIRI *n* (%)**	**FOLFIRI alone *n* (%)**	***P***
*EQ-5D HSI (*n)	279	289	0.010	262	265	0.017
Early dropout	145 (52.0)	181 (62.6)		158 (60.3)	186 (70.2)	
Late dropout/completer	134 (48.0)	108 (37.4)		104(39.7)	79 (29.8)	
						
*EQ-5D VAS (*n)	278	285	0.050	257	259	0.008
Early dropout	148 (53.2)	175 (61.4)		153 (59.5)	183 (70.7)	
Late dropout/completer	130 (46.8)	110 (38.6)		104 (40.5)	76 (29.3)	

Abbreviations: EQ-5D=EuroQoL-5 dimensions; FOLFIRI=fluorouracil, leucovorin and irinotecan; FOLFOX=fluorouracil, leucovorin and oxaliplatin; HSI=Health State Index; VAS=Visual Analogue Scale.

Note: early drop out is defined as study dropout on or before week 28 for the first-line trial and week 20 for the second-line trial. *P-*value is derived from a *χ*^2^-test of association.

**Table 4:** LSM differences in change from baseline in the EQ-5D HSI and EQ-5D VAS scores between (**A**) panitumumab + FOLFOX4 *vs* FOLFOX4 alone^a^ (**B**) panitumumab + FOLFIRI *vs* FOLFIRI alone^b^, using pattern mixture models

**(A)**						
	**EQ-5D HIS**			**EQ-5D VAS**		
Dropout group	**Panitumumb + FOLFOX4 (*n*=279)**	**FOLFOX4 (*n*=289)**	**Difference**	**Panitumumb + FOLFOX4 (*n*=278)**	**FOLFOX4 (*n*=285)**	**Difference**
Late/completer	0.058	0.062	−0.004	4.008	4.392	−0.384
(95% CI)	(0.032, 0.084)	(0.033, 0.091)	(−0.043, 0.035)	(1.677, 6.339)	(1.887, 6.898)	(−3.799, 3.030)
						
Early	−0.006	0.014	−0.020	−0.873	0.795	−1.668
(95% CI)	(−0.030, −0.018)	(−0.008, 0.036)	(−0.053, 0.012)	(−2.872, 1.125)	(−1.057, 2.646)	(−4.390, 1.054)
						
**(B)**						
	**EQ-5D HIS**			**EQ-5D VAS**		
**Dropout group**	**Panitumumb + FOLFIRI (*n*=262)**	**FOLFIRI (*n*=265)**	**Difference**	**Panitumumb + FOLFIRI (*n*=257)**	**FOLFIRI (*n*=259)**	**Difference**
Late/completer	0.023	0.025	−0.002	1.575	3.310	−1.735
(95% CI)	(−0.008, 0.053)	(−0.011, 0.060)	(−0.048, 0.044)	(−0.477, 3.627)	(0.875, 5.745)	(−4.913, 1.443)
						
Early	−0.059	−0.008	−0.051	−3.675	−1.866	−1.809
(95% CI)	(−0.084, −0.034)	(−0.031, 0.015)	(−0.085, −0.017)	(−5.349, −2.002)	(−3.406, −0.326)	(−4.090, 0.471)

Abbreviations: CI=confidence interval; EQ-5D=EuroQoL-5 dimensions; FOLFIRI=fluorouracil, leucovorin and irinotecan; FOLFOX=fluorouracil, leucovorin and oxaliplatin; HSI=Health State Index; LSM=least squares mean; VAS=Visual Analogue Scale.

Early drop out is defined as study dropout on or before ^a^week 28 in the first-line trial; ^b^week 20 in the second-line trial.

**Table 5:** (**A**) Baseline and LSM change and (**B**) LSM differences in change from baseline in the EQ-5D HSI and EQ-5D VAS scores by severity level of skin toxicity in the first- and second-line trials

**(A)**				
	**First-line trial**		**Second-line trial**	
**Severity level of skin toxicities**	**EQ-5D HSI**	**EQ-5D VAS**	**EQ-5D HSI**	**EQ-5D VAS**
*None/mild*				
Baseline	0.733	73.6	0.751	71.9
LSM change	−0.001	1.7	−0.076	−3.3
				
*Moderate*				
Baseline	0.780	73.9	0.783	73.0
LSM change	0.041	2.9	0.001	−0.7
				
*Severe*				
Baseline	0.794	74.5	0.772	74.1
LSM change	0.016	−0.5	−0.019	−1.6
				
**(B)**				
	**First-line trial**		**Second-line trial**	
**Difference between groups**	**EQ-5D HSI**	**EQ-5D VAS**	**EQ-5D HSI**	**EQ-5D VAS**
Moderate *vs* none/mild	0.042	1.206	0.077	2.571
(95% CI)	(−0.012, 0.095)	(−3.309, 5.720)	(0.014, 0.140)	(−1.645, 6.786)
				
Severe *vs* none/mild	0.017	−2.261	0.056	1.698
(95% CI)	(−0.038, 0.071)	(−6.869, 2.347)	(−0.003, 0.116)	(−2.349, 5.746)

Abbreviations: CI=confidence interval; EQ-5D=EuroQoL-5 dimensions; HSI=Health State Index; LSM=least squares mean; VAS=Visual Analogue Scale.

## References

[bib1] Agero AL, Dusza SW, Benvenuto-Andrade C, Busam KJ, Myskowski P, Halpern AC (2006) Dermatologic side effects associated with the epidermal growth factor receptors inhibitors. J Am Acad Dermatol 55: 657–670 17010747 10.1016/j.jaad.2005.10.010

[bib2] Amado R, Wolf M, Peeters M, Van Cutsem E, Siena S, Freeman DJ, Juan T, Sikorski R, Suggs S, Radinsky R, Patterson SD, Chang DD (2008) Wild-type *KRAS* is required for panitumumab efficacy in patients with metastatic colorectal cancer. J Clin Oncol 26: 1626–1634 18316791 10.1200/JCO.2007.14.7116

[bib3] Andreis F, Rizzi A, Mosconi P, Braun C, Rota L, Meriggi F, Mazzocchi M, Zaniboni A (2010) Quality of life in colon cancer patients with skin side effects: preliminary results from a monocentric cross sectional study. Health Qual Life Outcomes 8: 40 20398332 10.1186/1477-7525-8-40 PMC2861022

[bib4] Bokemeyer C, Bondarenko I, Makhson A, Hartmann JT, Aparicio J, de Braud F, Donea S, Ludwig H, Schuh G, Stroh C, Loos AH, Zubel A, Koralewski P (2009) Fluorouracil, leucovorin, and oxaliplatin with and without cetuximab in the first-line treatment of metastatic colorectal cancer. J Clin Oncol 27: 663–671 19114683 10.1200/JCO.2008.20.8397

[bib5] Byrne C, Griffin A, Blazeby J, Conroy T, Efficace F (2007) Health-related quality of life as a valid outcome in the treatment of advanced colorectal cancer. Eur J Surg Oncol 33: S95–S104 18039559 10.1016/j.ejso.2007.10.003

[bib6] Cella DF (1995) Measuring quality of life in palliative care. Semin Oncol 22: 73–81 7537908

[bib7] Dolan P (1997) Modeling valuations for EuroQol health states. Med Care 35: 1095–1108 9366889 10.1097/00005650-199711000-00002

[bib8] Douillard JY, Siena S, Caissdy J, Tabernero J, Burkes R, Barugel M, Humblet Y, Bodoky G, Cunningham D, Jassem J, Rivera F, Kocákova I, Ruff P, Blasin¨ska-Morawiec M, S˘makal M, Canon JL, Rother M, Oliner KS, Wolf M, Gansert J (2010) Randomized, phase III trial of panitumumab with infusional fluorouracil, leucovorin, and oxaliplatin (FOLFOX4) verus FOLFOX4 alone as a first-line treatment in patients with previously untreated metastatic colorectal cancer: the PRIME study. J Clin Oncol 28: 4697–4705 20921465 10.1200/JCO.2009.27.4860

[bib9] Folprecht G, Nowacki M, Lang I, Cascinu S, Shchepotin I, Maurel J, Rougier P, Cunningham D, Zubel A, Van Cutsem E (2009) Cetuximab plus FOLFIRI first-line in patients (pts) with metastatic colorectal cancer (mCRC): a quality of life (QoL) analysis of the CRYSTAL trial. J Clin Oncol 127: 15s Abstract 4076

[bib10] Hurwitz H, Fehrenbacher L, Novotny W, Cartwright T, Hainsworth J, Heim W, Berlin J, Baron A, Griffling S, Holmgren E, Ferrara N, Fyfe F, Rodgers B, Ross R, Kabbinavar F (2004) Bevacizumab plus irinotecan, fluorouracil, and leucovorin for metastatic colorectal cancer. N Engl J Med 350: 2335–2342 15175435 10.1056/NEJMoa032691

[bib11] Kabbinavar FF, Wallace JF, Holmgren E, Yi J, Cella D, Yost KJ, Hurwitz HI (2008) Health-related quality of life impact of bevacizumab when combined with irinotecan, 5-fluorouracil, and leucovorin or 5-fluorouracil and leucovorin for metastatic colorectal cancer. Oncologist 13: 1021–1029 18776057 10.1634/theoncologist.2008-0003

[bib12] Karapetis CS, Khambat-Ford S, Jonker DJ, O'Callaghan CJ, Tu D, Tebbutt NC, Simes RJ, Chalchal H, Shapiro JD, Robitaille S, Price TJ, Shepherd L, Au HJ, Langer C, Moore MJ, Zalcberg JR (2008) K-ras mutations and benefits from cetuximab in advanced colorectal cancer. N Eng J Med 359: 1757–1765 10.1056/NEJMoa0804385 18946061

[bib13] Krabbe PF, Peerenboom L, Langenhoff BS, Ruers TJ (2004) Responsiveness of the generic EQ-5D summary measure compared to the disease-specific EORTC QLQ C-30. Qual Life Res 13: 1247–1253 15473503 10.1023/B:QURE.0000037498.00754.b8

[bib14] National Cancer Institute, National Institutes of Health, Department of Health and Human Services (2006) Cancer Therapy Evaluation Program: Common Terminology Criteria for Adverse Events, version 3.0. Bethesda, MD

[bib15] Odom D, Barber B, Bennett L, Peeters M, Zhao Z, Kaye J, Wolf M, Wiezorek J (2011) Health-related quality of life and colorectal cancer-specific symptoms in patients with chemotherapy-refractory metastatic disease treated with panitumumab. Int J Colorectal Dis 26: 173–181 21190026 10.1007/s00384-010-1112-5 PMC3024508

[bib16] Peeters M, Price TJ, Cervantes A, Sobrero AF, Ducreux M, Hotko Y, André T, Chan E, Lordick F, Punt CJ, Strickland AH, Wilson G, Ciuleanu TE, Roman L, Van Cutsem E, Tzekova V, Collins S, Oliner KS, Rong A, Gansert J (2010) Randomized phase III study of panitumumab with FOLFIRI *vs* FOLFIRI alone as a second-line treatment in patients with metastatic colorectal cancer. J Clin Oncol 28: 4706–4713 20921462 10.1200/JCO.2009.27.6055

[bib17] Petrou S, Hockley C (2005) An investigation into the empirical validity of the EQ-5D and SF-6D based on hypothetical preferences in a general population. Health Econ 14: 1169–1189 15942981 10.1002/hec.1006

[bib18] Pickard AS, Neary MP, Cella D (2007a) Estimation of minimally important differences in EQ-5D utility and VAS scores in cancer. Health Qual Life Outcomes 5: 70 18154669 10.1186/1477-7525-5-70 PMC2248572

[bib19] Pickard AS, Wilke CT, Lin HW, Lloyd A (2007b) Health utilities using the EQ-5D in studies of cancer. Pharmacoeconomics 25: 365–384 17488136 10.2165/00019053-200725050-00002

[bib20] Saltz LB, Clarke S, Díaz-Rubio E, Scheithauer W, Figer A, Wong R, Koski S, Lichinitser M, Yang TS, Rivera F, Couture F, Sirzén F, Cassidy J (2008) Bevacizumab in combination with oxaliplatin-based chemotherapy as first-line therapy in metastatic colorectal cancer: a randomized phase III study. J Clin Oncol 26: 2013–2019 18421054 10.1200/JCO.2007.14.9930

[bib21] Siddiqui O, Hung H, O'Neill R (2009) MMRM *vs* LOCF: a comphrensive comparison based on simulation study and 25 NDA datasets. J Biopharm Stat 19: 227–246 19212876 10.1080/10543400802609797

[bib22] Siena S, Peeters M, van Cutsem E, Humblet Y, Conte P, Bajetta E, Comandini D, Bodoky G, Van Hazel G, Salek T, Wolf M, Devercelli G, Woolley M, Amado RG (2007) Association of progression-free survival with patient-reported outcomes and survival: results from a randomised phase 3 trial of panitumumab. Br J Cancer 97: 1469–1474 18040272 10.1038/sj.bjc.6604053 PMC2360255

[bib23] Tebbutt NC, Wilson K, Gebski VJ, Cummins MM, van Hazel GA, Robinson B, Broad A, Ganju V, Ackland SP, Forgeson G, Cunningham D, Saunders MP, Stockler MR, Chua Y, Zalcberg JR, Simes RJ, Price TJ (2010) Capecitabine, bevacizumab, and mitomycin in first-line treatment of metastatic colorectal cancer: results of the Australasian Gastrointestinal Trials Group Randomised Phase III MAX Study. J Clin Oncol 28: 3191–3198 20516443 10.1200/JCO.2009.27.7723

[bib24] Van Cutsem E, Köhne CH, Hitre E, Zaluski J, Chang Chien CR, Makhson A, D'Haens G, Pintér T, Lim R, Bodoky G, Roh JK, Folprecht G, Ruff P, Stroh C, Teipar S, Schlichting M, Nippgen J, Rougier P (2009) Cetuximab and chemotherapy as initial treatment for metastatatic colorectal cancer. N Engl J Med 360: 1408–1417 19339720 10.1056/NEJMoa0805019

[bib25] Van Cutsem E, Nordlinger B, Cervantes A, ESMO Guidelines Working Group (2010) Advanced colorectal cancer: ESMO Clinical Practice Guidelines for treatment. Ann Oncol 21: v93–v97 20555112 10.1093/annonc/mdq222

